# Distinct patterns of brain atrophy in Genetic Frontotemporal Dementia Initiative (GENFI) cohort revealed by visual rating scales

**DOI:** 10.1186/s13195-018-0376-9

**Published:** 2018-05-24

**Authors:** Giorgio G. Fumagalli, Paola Basilico, Andrea Arighi, Martina Bocchetta, Katrina M. Dick, David M. Cash, Sophie Harding, Matteo Mercurio, Chiara Fenoglio, Anna M. Pietroboni, Laura Ghezzi, John van Swieten, Barbara Borroni, Alexandre de Mendonça, Mario Masellis, Maria C. Tartaglia, James B. Rowe, Caroline Graff, Fabrizio Tagliavini, Giovanni B. Frisoni, Robert Laforce, Elizabeth Finger, Sandro Sorbi, Elio Scarpini, Jonathan D. Rohrer, Daniela Galimberti, on behalf of the Genetic FTD Initiative (GENFI), Christin Andersson, Silvana Archetti, Luisa Benussi, Giuliano Binetti, Sandra Black, Maura Cosseddu, Marie Fallström, Carlos Ferreira, Nick C. Fox, Morris Freedman, Stefano Gazzina, Roberta Ghidoni, Marina Grisoli, Vesna Jelic, Lize Jiskoot, Ron Keren, Gemma Lombardi, Carolina Maruta, Simon Mead, Lieke Meeter, Rick van Minkelen, Benedetta Nacmias, Linn Öijerstedt, Sebastien Ourselin, Alessandro Padovani, Jessica Panman, Michela Pievani, Cristina Polito, Enrico Premi, Sara Prioni, Rosa Rademakers, Veronica Redaelli, Ekaterina Rogaeva, Giacomina Rossi, Martin N. Rossor, David Tang-Wai, David L. Thomas, Hakan Thonberg, Pietro Tiraboschi, Ana Verdelho, Jason D. Warren

**Affiliations:** 1 Department of Pathophysiology and Transplantation, “Dino Ferrari” Center, University of Milan, Milan, Italy; 2 Fondazione Cà Granda, IRCCS Ospedale Maggiore Policlinico, Milan, Italy; 3 Department of Neurosciences, Psychology, Drug Research and Child Health (NEUROFARBA), University of Florence, Florence, Italy; 4 Dementia Research Centre, Department of Neurodegenerative Disease, UCL Institute of Neurology, Queen Square, London, UK; 5 Erasmus Medical Center, Rotterdam, The Netherlands; 6 University of Brescia, Brescia, Italy; 7 Faculdade de Medicina, Universidade de Lisboa, Lisbon, Portugal; 8 Cognitive Neurology Research Unit, Sunnybrook Health Sciences Centre, Hurvitz Brain Sciences Research Program, Sunnybrook Research Institute, Department of Medicine, University of Toronto, Toronto, ON Canada; 9 Tanz Centre for Research in Neurodegenerative Diseases, University of Toronto, Toronto, ON, Canada; 10 University of Cambridge, Cambridge, UK; 11 Center for Alzheimer Research, Division of Neurogeriatrics, Department of Neurobiology, Care Sciences and Society, Karolinska Institutet, Stockholm, Sweden; 12 Department of Geriatric Medicine, Karolinska University Hospital, Stockholm, Sweden; 13 Istituto Neurologico Carlo Besta, Milan, Italy; 14 IRCCS San Giovanni di Dio Fatebenefratelli, Brescia, Italy; 15 Université Laval, Quebec, QC Canada; 16 University of Western Ontario, London, ON Canada; 17 IRCCS Don Gnocchi, Florence, Italy

**Keywords:** Frontotemporal dementia, Genetics, MRI, Visual rating

## Abstract

**Background:**

In patients with frontotemporal dementia, it has been shown that brain atrophy occurs earliest in the anterior cingulate, insula and frontal lobes. We used visual rating scales to investigate whether identifying atrophy in these areas may be helpful in distinguishing symptomatic patients carrying different causal mutations in the microtubule-associated protein tau (*MAPT*), progranulin (*GRN*) and chromosome 9 open reading frame *(C9ORF72)* genes. We also analysed asymptomatic carriers to see whether it was possible to visually identify brain atrophy before the appearance of symptoms.

**Methods:**

Magnetic resonance imaging of 343 subjects (63 symptomatic mutation carriers, 132 presymptomatic mutation carriers and 148 control subjects) from the Genetic Frontotemporal Dementia Initiative study were analysed by two trained raters using a protocol of six visual rating scales that identified atrophy in key regions of the brain (orbitofrontal, anterior cingulate, frontoinsula, anterior and medial temporal lobes and posterior cortical areas).

**Results:**

Intra- and interrater agreement were greater than 0.73 for all the scales. Voxel-based morphometric analysis demonstrated a strong correlation between the visual rating scale scores and grey matter atrophy in the same region for each of the scales. Typical patterns of atrophy were identified: symmetric anterior and medial temporal lobe involvement for *MAPT*, asymmetric frontal and parietal loss for *GRN*, and a more widespread pattern for *C9ORF72*. Presymptomatic *MAPT* carriers showed greater atrophy in the medial temporal region than control subjects, but the visual rating scales could not identify presymptomatic atrophy in *GRN* or *C9ORF72* carriers.

**Conclusions:**

These simple-to-use and reproducible scales may be useful tools in the clinical setting for the discrimination of different mutations of frontotemporal dementia, and they may even help to identify atrophy prior to onset in those with *MAPT* mutations.

## Background

Frontotemporal dementia (FTD) is a neurodegenerative disease characterized clinically by changes in behaviour or language. Up to one-third of cases are caused by mutations in one of three major causal genes identified so far: microtubule-associated protein tau (*MAPT*), progranulin (*GRN*) and chromosome 9 open reading frame 72 (*C9ORF72*) [1–3].

Structural neuroimaging is recommended as part of the clinical evaluation in all patients with suspected dementia and forms part of the diagnostic criteria of FTD [4, 5]. Previous studies have shown that each mutation has a distinct pattern of atrophy: Mutations in *MAPT* have been associated with atrophy predominantly in the anteromedial temporal lobes [6, 7], whereas mutations in *GRN* are associated with an asymmetric pattern of atrophy that involves the frontal, temporal and parietal lobes [6, 8]; *C9ORF72* mutation carriers have relatively widespread cortical atrophy, including posterior areas [1, 9, 10]. However, such studies have relied on volumetric ROIs or voxel-wise analyses that are difficult to translate into routine clinical practice, where visual evaluation remains the primary diagnostic method [11].

To provide reliable identification and interpretation of imaging findings, different visual rating scales have been developed over time (*see* [12] for a review). Recently, in a multi-centre pathologically confirmed series, we have shown that visual rating scales can improve the accuracy of clinical diagnosis of different dementias [13]. However, only one study of visual rating scales has investigated the genetic forms of FTD so far [14], and only in symptomatic patients in one genetic subtype, *C9ORF72*. The objective of the present study was therefore to determine specific visual patterns of atrophy in genetic FTD, in both symptomatic and presymptomatic mutation carriers, and in all three of the major genetic forms: *GRN*, *MAPT* and *C9ORF72*.

## Methods

### Participants

Subjects were recruited from the Genetic Frontotemporal Dementia Initiative (GENFI) study, which in the first phase consisted of 13 centres in the United Kingdom, Canada, Italy, The Netherlands, Sweden and Portugal. We included participants who were either known carriers of a pathogenic mutation in *MAPT*, *GRN* or *C9ORF72,* or family members at risk of carrying a mutation. In the at-risk group, those who had positive genetic testing were included in the pre-symptomatic group, and those that had negative genetic testing were included in the control group. In this way control subjects shared a similar environmental background but differed from carriers only for the absence of a pathogenic mutation in *MAPT*, *GRN* or *C9ORF72*. Participants were genotyped at their local site. Between January 2012 and April 2015, we enrolled 365 subjects, 343 of whom had a usable volumetric T1-weighted magnetic resonance imaging (MRI) scan. Local ethics committees at each site approved the study, and all participants provided written informed consent at enrolment.

### Procedures

All participants underwent a standardized clinical assessment and a full neuropsychological battery (for details, *see* Rohrer et al., 2015 [3]). Participants were scanned at their local site on scanners from three different manufacturers (Philips Healthcare, GE Healthcare Life Sciences, Siemens Healthcare Diagnostics). Magnetic field strength was 3 T for 295 scans (86%) and 1.5 T for 48 scans (14%). The protocol, designed to match across scanners as much as possible, included a volumetric T1-weighted MRI scan.

### Visual rating scales

A protocol of 6 visual rating scales was applied in the cohort by two raters (GGF and PB), blinded to all clinical and demographic information, after a training set of 15 scans that included 5 cases with a clinical diagnosis of behavioural variant FTD, 5 with primary progressive aphasia and 5 control subjects. The training set was not included in the main analysis. The protocol made use of previously validated scales with particular attention to areas known to show atrophy in FTD [13]. The following scales were chosen: orbitofrontal (OF), anterior cingulate (AC), frontoinsula (FI), anterior temporal (AT), medial temporal (MTA) and posterior (PA). With the OF, AC and FI scales, we looked at the widening of a single sulcus. Raters looked at the olfactory sulcus for the OF region, the anterior part of cingulate sulcus for AC region and the circular sulcus for the FI region. For all three scales, a four-part grading system was used: grade 0, representing no atrophy (no cerebrospinal fluid [CSF] visible within the sulcus); grade 1, mild widening of the sulcus (CSF just becomes visible); grade 2, moderate widening; and grade 3, severe widening (with the sulcus assuming a triangular shape). In order to ensure that the same areas were being reviewed on each scan, specific anatomical landmarks were used. The olfactory and cingulate sulci were reviewed in the coronal plane on the most anterior slice in which the corpus callosum was visible, whilst the circular sulcus was assessed also in the coronal plane, on the most anterior slice in which the anterior commissure was visible, as well as the two slices immediately posterior to this [13]. The AT scale looked at the aspect of the temporal pole in coronal view, using a 5-point system: grade 0 representing normal appearances, grade 1 only slight prominence of anterior temporal sulci, grade 2 definite widening of the temporal sulci, grade 3 severe atrophy and ribbon-like nature of the gyri, and grade 4 a simple linear profile of the temporal pole [15, 16]. The MTA is a 5-point graded scale that looks at the medial temporal lobe in coronal view: grade 0 is normal; grade 1 a widened choroidal fissure; grade 2 an increased widening of the choroidal fissure, widening of temporal horn and opening of other sulci; grade 3 pronounced volume loss of the hippocampus; and grade 4 end-stage atrophy [17]. The last scale used was PA, a 4-point scale evaluating posterior cortical atrophy using three views (coronal, axial and sagittal): grade 0 representing closed posterior cingulate and parieto-occipital sulci; grade 1 mild widening of the posterior cingulate and parieto-occipital sulci, with mild atrophy of the parietal lobes and precuneus; grade 2 substantial widening of the posterior cingulate and parieto-occipital sulcus, with substantial atrophy of the parietal lobes and precuneus; and grade 3 end-stage atrophy with evident widening of both sulci and knife-blade atrophy of the parietal lobes and precuneus [18].

The software used for the visualization of the images was MRIcron [19]. Images were rated in native space, in keeping with standard clinical reads. To aid rating consistency, reference images for each scale were provided to the raters. Right and left sides were assessed separately. The mean score of the two raters for each subject was calculated by averaging a combined right- and left-sided score in each rating scale. An asymmetry index was calculated as the sum of the absolute differences between the two sides for each scale. The raters re-rated a subset of 35 subjects randomly chosen in the main group to calculate intra-rater reliability.

To explore the relationship between each rating scale and the pattern of grey matter (GM) density, voxel-based morphometric analysis was performed using Statistical Parametric Mapping 12 [12]. T1-weighted images were normalized and segmented into GM, white matter and CSF probability maps by using standard procedures and the fast-diffeomorphic image registration (DARTEL) algorithm [20]. GM segments were affine-transformed into the MNI (Montreal Neurological Institute) space, modulated and smoothed using a Gaussian kernel with 6-mm FWHM before analysis. The GM tissue maps were fitted to a multiple regression model to identify the correlations with the six rating scales (OF, AC, FI, AT, MTA, PA). Age, sex and total intracranial volume were entered as covariates. The family-wise error rate for multiple comparisons correction was set at 0.05.

### Statistical analysis

All the statistical analyses were performed using IBM SPSS Statistics version 22 for Windows software (IBM, Armonk, NY, USA). Differences in age and education were assessed with the *t* test, and differences in sex were evaluated with the χ^2^ test. Differences in the visual rating scale scores between groups were assessed using the Mann-Whitney *U* test. Inter- and Intra-rater reliability of each rating scale was determined using a two-way random, absolute, single-measure intra-class correlation coefficient (ICC).

## Results

### Demographics

The cohort consisted of 343 subjects, including 132 presymptomatic and 63 symptomatic individuals as well as 148 control subjects (*see* Table 1). Symptomatic subjects were older than control subjects, independently of the mutation status. Moreover, the *MAPT* symptomatic carriers were younger than *GRN* and *C9ORF72* symptomatic carriers, as were *MAPT* presymptomatic carriers compared with the other two groups of asymptomatic carriers. Regarding sex, symptomatic *MAPT* and *C9ORF72* carriers were significantly different (*p* < 0.05) from control subjects and *GRN* symptomatic carriers.

**Table 1:** Demographic data and visual rating scores

	Control subjects	*GRN*	*C9ORF72*	*MAPT*	Total	*GRN*	*C9ORF72*	*MAPT*	Total	Significance
		Presymptomatic				Symptomatic				
Number	148	66	42	24	132	17	31	15	63	
Age	48.86 (14.32)	49.55 (10.90)	44.66 (11.49)	38.70 (8.80)	46.02 (11.42)	63.96 (5.70)	65.92 (7.61)	57.17 (8.21)	63.31 (8.02)	^a^*^,b^*^,c^*^,d,e,g^*
Sex	90 M	41 M	25 M	14 M	80 M	11 M	9 M	4 M	24 M	^c,d,e,f^
	58 F	25 F	17 F	10 F	52 F	6 F	22 F	11 F	39 F	
OF	0.19 (0.33)	0.20 (0.35)	0.33 (0.55)	0.28 (0.70)	0.26 (0.50)	1.99 (0.55)	1.54 (0.72)	1.43 (0.70)	1.63 (0.70)	^b^*^,c^*^,d^*^,e,f^
AC	0.35 (0.37)	0.46 (0.49)	0.45 (0.49)	0.38 (0.65)	0.44 (0.52)	2.09 (0.69)	1.65 (0.69)	1.35 (0.52)	1.69 (0.70)	^b^*^,c^*^,d^*^,f^
AT	0.22 (0.35)	0.30 (0.38)	0.30 (0.48)	0.31 (0.72)	0.30 (0.48)	1.53 (0.57)	1.44 (0.71)	2.38 (0.93)	1.69 (0.82)	^b^*^,c^*^,d^*^,f,g^
FI	0.61 (0.47)	0.64 (0.47)	0.66 (0.64)	0.49 (0.55)	0.62 (0.54)	2.24 (0.42)	2.10 (0.50)	1.80 (0.52)	2.07 (0.50)	^b^*^,c^*^,d^*^,f^
MTA	0.28 (0.41)	0.31 (0.40)	0.45 (0.74)	0.51 (0.74)	0.39 (0.60)	1.40 (0.64)	1.82 (1.10)	2.60 (1.28)	1.89 (1.12)	^a,b^*^,c^*^,d^*^,f^
PA	0.36 (0.52)	0.33 (0.52)	0.42 (0.59)	0.18 (0.25)	0.33 (0.51)	1.79 (0.77)	1.66 (0.72)	0.77 (0.55)	1.48 (0.80)	^b^*^,c^*^,d,f^*^,g^*
ASYMM	1.49 (0.90)	1.79 (1.20)	1.62 (1.08)	1.50 (0.92)	1.68 (1.11)	4.41 (2.00)	2.92 (1.23)	2.20 (0.88)	3.15 (1.62)	^b^*^,c^*^,d,e,f^*^,g^

*Abbreviations: GRN* Progranulin, *C9ORF72* Chromosome 9 open reading frame 72, *MAPT* Microtubule-associated protein tau, *OF* Orbitofrontal rating scale, *AC* Anterior cingulate rating scale, *AT* Anterior temporal rating scale, *FI* Frontoinsula rating scale, *MTA* Medial temporal atrophy rating scale, *PA* Posterior atrophy rating scale, *ASYMM* Asymmetry index

Data are reported as mean (SD). The *t* test was used for age and education, the χ^2^ was used for sex, and the Mann-Whitney *U* test was used for visual rating scales

^a^Controls vs Presymtomatic *MAPT*

^b^Controls vs Symptomatic *GRN*

^c^Controls vs Symptomatic *C9ORF72*

^d^Controls vs Symptomatic *MAPT*

^e^Symptomatic *GRN* vs Symptomatic *MAPT*

^f^Symptomatic *GRN* vs Symptomatic *C9ORF72*

^g^Symptomatic *C9ORF72* vs Symptomatic *MAPT*

* *P* < 0.001; otherwise, *P* < 0.05

### Intra- and inter-rater reliability

All the scales demonstrated good inter-rater reliability (ICC > 0.73) (*see* Table 2), with the MTA scale performing best overall. Considering the intra-rater scores, rater 1 ICCs were greater than 0.82 for all the scales, whereas rater 2 had scores greater than 0.89 for all the scales.

**Table 2:** Intra and inter rater agreement scores

		OF	AC	AT	FI	MTA	PA
Interrater	Raters 1–2	0.82	0.74	0.77	0.75	0.88	0.73
Intrarater	Rater 1	0.89	0.82	0.95	0.82	0.90	0.93
	Rater 2	0.97	0.90	0.96	0.91	0.96	0.89

*Abbreviations: OF* Orbitofrontal rating scale, *AC* Anterior cingulate rating scale, *AT* Anterior temporal rating scale, *FI* Frontoinsula rating scale, *MTA* Medial temporal atrophy rating scale, *PA* Posterior atrophy rating scale

Inter- and intra-rater agreement intraclass correlation coefficient score for each visual rating scale is shown

### Mean visual rating scores

**Fig. 1:**
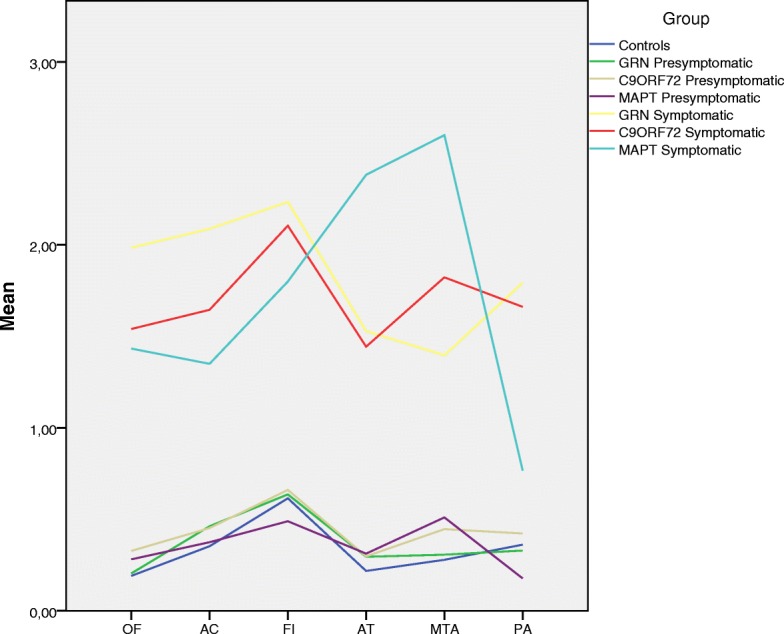
Mean score of each visual rating scale in each group

All the scales and the asymmetric index were significantly higher in the three symptomatic groups than for the respective control subjects (Table 1, Fig. 1). Symptomatic carriers of *MAPT* had higher scores in the AT region (2.38) than the other two groups (*GRN*, 1.53; *p* = 0.002; *C9ORF72*, 1.44; *p* = 0.001) and in the MTA scale (2.60) than *GRN* (1.40; *p* = 0.005), with a trend in comparison with *C9ORF72* (1.82; *p* = 0.061). By contrast, symptomatic carriers of *GRN* obtained higher scores in the OF scale (1.99) than the other two groups (*MAPT*, 1.43; *p* = 0.016; *C9ORF72*, 1.54; *p* = 0.043) and in the AC (2.09), FI (2.24), and PA (1.79) scales compared with *MAPT* (AC, 1.35; *p* = 0.004; FI, 1.80; *p* = 0.014; PA, 0.77; *p* < 0.001) but not *C9ORF72*. *GRN* symptomatic carriers also showed the highest asymmetry index scores (4.41) compared with the other two groups (*C9ORF72*, 2.92; *p* = 0.009; *MAPT*, 2.20; *p* < 0.001), with *C9ORF72* showing a significantly higher index than *MAPT* (*p* = 0.036). Symptomatic carriers of *C9ORF72* scored higher than *MAPT* only in the PA scale (*C9ORF72*, 1.66; *MAPT*, 0.77; *p* < 0.001).

In the presymptomatic group, *MAPT* carriers scored significantly higher than the control subjects in the MTA scale (*MAPT*, 0.51; CON, 0.28; *p* = 0.029) but not the other scales. No differences were found in the comparison of the other two presymptomatic groups (*GRN* and *C9ORF72*) with control subjects. There were also no differences between presymptomatic groups in terms of scores on the visual scales or on the asymmetry index.

### Voxel-based morphometric analysis

**Fig. 2:**
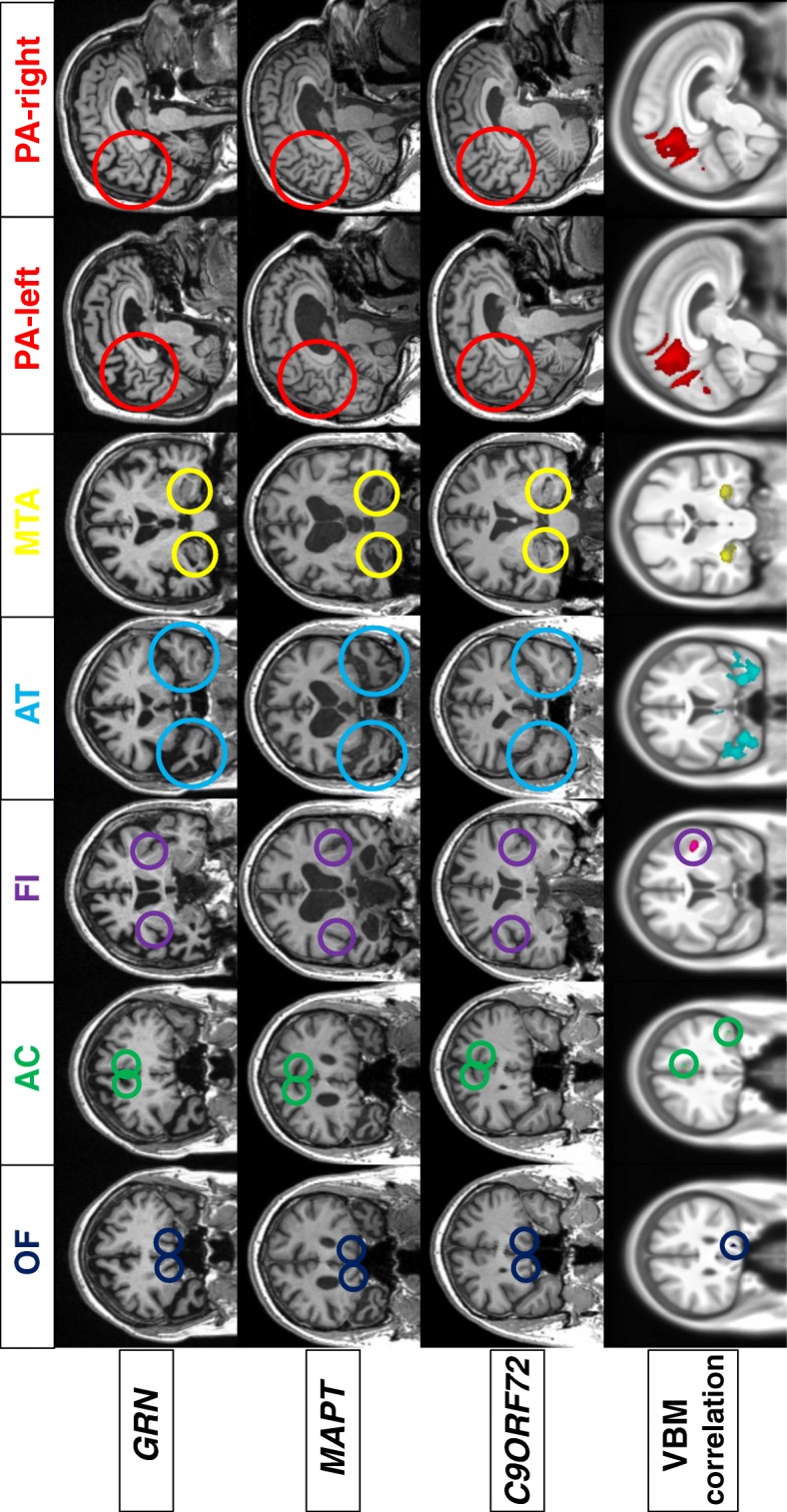
Representative coronal (for all scales except posterior atrophy scale) and sagittal (posterior atrophy scale) T1-weighted magnetic resonance images of symptomatic carriers of *GRN*, *MAPT*, and *C9ORF72* mutations (top three rows). Voxel-based morphometric analysis showing areas of significant negative correlation between the scales and grey matter. Maps showing z-scores were rendered on a study-specific template in MNI (Montreal Neurological Institute) space (bottom row). Images are shown with the left hemisphere on the left side of the figure

The voxel-based morphometric analysis revealed a negative correlation of each visual rating scale score with an area of GM atrophy in the same (expected) region (*see* Fig. 2). No positive correlations were found.

## Discussion

Using simply applied visual rating scales, we have identified typical patterns of atrophy for each group of mutation carriers: anterior and medial temporal for *MAPT*, asymmetric frontal (orbitofrontal, cingulate, frontoinsular) and parietal for *GRN* and widespread for *C9ORF72* (*see* Fig. 2). This is consistent with previous studies on patients with genetic FTD using voxel-based morphometry [6, 9, 10], even with the limitations of a semiquantitative assessment of atrophy. We also validated the visual rating scales using voxel-based morphometry, with each scale correlating with the specific brain region that the scale was designed to assess.

Only one previous study has investigated visual rating scales in genetic FTD: Devenney et al. used seven visual rating scales to describe the features of symptomatic *C9ORF72* carriers, but they did not find any statistical differences between *C9ORF72* and control subjects; they observed only a trend toward greater precuneus atrophy [14].

It has recently been demonstrated that GM changes can be identified years before the expected onset of symptoms in adults at risk of genetic FTD [3]. In particular, in individuals with *MAPT* mutations, atrophy was first noted in the hippocampus and amygdala, followed by the temporal lobe and later the insula; in *GRN* mutation carriers, differences started in the insula, followed by the temporal and parietal lobes and thereafter the striatum; in the *C9ORF72* group, changes were found very early in subcortical areas, the insula and the occipital cortex, then the frontal and temporal lobes and subsequently the cerebellum. This differential neuroanatomical involvement within the three genetic groups is likely to represent differently vulnerable large-scale neural networks, with pathological protein spread through those networks as the disease progresses. However, the underlying biology of this differential vulnerability is not yet understood. In our study, we demonstrated that presymptomatic *MAPT* carriers without symptoms had more medial temporal lobe atrophy than control subjects. However, we could not find any presymptomatic difference for individuals with *GRN* or *C9ORF72* mutations. There may be multiple reasons for this, including the nature of the cohort (with a number of cases far from expected onset), the pattern of atrophy (involvement of areas such as striatum, thalamus and cerebellum not identified by such scales), and the lack of sensitivity of the technique (which is likely to be more so for some regions than others).

Asymmetric atrophy is confirmed as one of the main features of *GRN* patients, but we could not find it in presymptomatic *GRN* carriers [3], probably because the changes are mild and are seen just a few years before the onset of the disease, whereas in our study we considered the group of presymptomatic cases as a whole, without stratifying by expected age at onset. Unexpectedly, we found *C9ORF72* patients to be more asymmetric than *MAPT*, although less than *GRN*. This is in contrast to previous studies that showed a relatively symmetric atrophy in frontal, temporal and parietal lobes in *C9ORF72* patients [9, 21, 22]. A possible explanation can be that visual rating scales measure sulcal opening, which can reflect not only the amount of cortical GM atrophy but also other factors, such as CSF or white matter, that can be different in *C9ORF72*. Nevertheless, our study’s aim was to replicate real-life visual assessment of MRI scans using scanners of different types and field strengths and not requiring any expensive software or time-consuming post-processing techniques. The raters adopted a naturalistic approach, independently identifying the slices for the rating. This can result in rating different slices, but the scores obtained by the two raters in terms of intra- and inter-rater reliability are in line with the literature or better in the case of MTA [23].

## Conclusions

We have demonstrated differences among groups of mutations using a simple-to-use, reproducible and validated set of visual rating scales. Patterns of atrophy can be useful to help differentiate these groups and help predict the presence of a gene mutation in subjects with FTD. Clinicians can integrate the information obtained using MRI data with clinical features (e.g., psychosis in *C9ORF72*) and family history to tailor an approach to genetic testing [9, 14]. Further studies of visual rating scales of other important regions (such as subcortical areas) may add to our findings in improving differentiation between different mutations in FTD.

## Appendix

***Members of the GENFI Consortium*** **:**

Christin Andersson, Department of Clinical Neuroscience, Karolinska Institutet, Stockholm, Sweden [christin.andersson@karolinska.se]

Silvana Archetti, Biotechnology Laboratory, Department of Diagnostics, Civic Hospital of Brescia, Brescia, Italy [archetti.s@tiscali.it]

Luisa Benussi, Istituto di Ricovero e Cura a Carattere Scientifico, Istituto Centro San Giovanni di Dio Fatebenefratelli, Brescia, Italy [lbenussi@fatebenefratelli.eu]

Giuliano Binetti, Istituto di Ricovero e Cura a Carattere Scientifico, Istituto Centro San Giovanni di Dio Fatebenefratelli, Brescia, Italy; [gbinetti@fatebenefratelli.eu].

Sandra Black, L.C. Campbell Cognitive Neurology Research Unit, Sunnybrook Research Institute, Toronto, ON, Canada [sandra.black@sunnybrook.ca]

Maura Cosseddu, Centre of Brain Aging, University of Brescia, Brescia, Italy [maura.cosseddu@gmail.com]

Marie Fallström, Department of Geriatric Medicine, Karolinska University Hospital, Stockholm, Sweden [marie.fallstrom@karolinska.se]

Carlos Ferreira, Instituto Ciências Nucleares Aplicadas à Saúde, Universidade de Coimbra, Coimbra, Portugal [c_dferreira@yahoo.com]

Nick C. Fox, Dementia Research Centre, UCL Institute of Neurology, London, UK [n.fox@ucl.ac.uk]

Morris Freedman, Division of Neurology, Baycrest Centre for Geriatric Care, University of Toronto, ON, Canada [mfreedman@baycrest.org]

Stefano Gazzina, Centre of Brain Aging, Neurology Unit, Department of Clinical and Experimental Sciences, University of Brescia, Brescia, Italy [stefanogazzina@alice.it]

Roberta Ghidoni, Istituto di Ricovero e Cura a Carattere Scientifico, Istituto Centro San Giovanni di Dio Fatebenefratelli, Brescia, Italy [rghidoni@fatebenefratelli.eu]

Marina Grisoli, Fondazione Istituto di Ricovero e Cura a Carattere Scientifico, Istituto Neurologico Carlo Besta, Milan, Italy [marina.grisoli@istituto-besta.it]

Vesna Jelic, Division of Clinical Geriatrics, Karolinska Institutet, Stockholm, Sweden [vesna.jelic@ki.se]

Lize Jiskoot, Department of Neurology, Erasmus Medical Center, Rotterdam, The Netherlands [l.c.jiskoot@erasmusmc.nl]

Ron Keren, University Health Network Memory Clinic, Toronto Western Hospital, Toronto, ON, Canada [ron.keren@uhn.ca]

Gemma Lombardi, Department of Neuroscience, Psychology, Drug Research and Child Health, University of Florence, Florence, Italy [gemmalomb@gmail.com]

Carolina Maruta, Lisbon Faculty of Medicine, Language Research Laboratory, Lisbon, Portugal [carolmaruta@gmail.com]

Simon Mead, MRC Prion Unit, Department of Neurodegenerative Disease, UCL Institute of Neurology, Queen Square, London, UK [s.mead@prion.ucl.ac.uk]

Lieke Meeter, Department of Neurology, Erasmus Medical Center, Rotterdam, The Netherlands [h.meeter@erasmusmc.nl]

Rick van Minkelen, Department of Clinical Genetics, Erasmus Medical Center, Rotterdam, The Netherlands [r.vanminkelen@erasmusmc.nl]

Benedetta Nacmias, Department of Neuroscience, Psychology, Drug Research and Child Health, University of Florence, Florence, Italy [benedetta.nacmias@unifi.it]

Linn Öijerstedt, Division of Neurogeriatrics, Karolinska Institutet, Stockholm, Sweden [linn.oijerstedt@ki.se]

Sebastien Ourselin, Centre for Medical Image Computing, University College London, London, UK [s.ourselin@ucl.ac.uk]

Alessandro Padovani, Neurology Unit, Department of Medical and Experimental Sciences, University of Brescia, Brescia, Italy [alessandro.padovani@unibs.it]

Jessica Panman, Department of Neurology, Erasmus Medical Center, Rotterdam, The Netherlands [j.panman@erasmusmc.nl]

Michela Pievani, Istituto di Ricovero e Cura a Carattere Scientifico, Istituto Centro San Giovanni di Dio Fatebenefratelli, Brescia, Italy [mpievani@fatebenefratelli.eu]

Cristina Polito, Department of Clinical Pathophysiology, University of Florence, Florence, Italy [cristina.polito@unifi.it]

Enrico Premi, Centre for Ageing Brain and Neurodegenerative Disorders, Neurology Unit, University of Brescia, Brescia, Italy [zedtower@gmail.com]

Sara Prioni, Fondazione Istituto di Ricovero e Cura a Carattere Scientifico, Istituto Neurologico Carlo Besta, Milan, Italy [sara.prioni@istituto-besta.it]

Rosa Rademakers [London, ON, Canada, geneticist], Department of Neurosciences, Mayo Clinic, Jacksonville, FL, USA [rademakers.rosa@mayo.edu]

Veronica Redaelli, Fondazione Istituto di Ricovero e Cura a Carattere Scientifico, Istituto Neurologico Carlo Besta, Milan, Italy [veronica.redaelli@istituto-besta.it]

Ekaterina Rogaeva, Tanz Centre for Research in Neurodegenerative Diseases, University of Toronto, Toronto, ON, Canada [ekaterina.rogaeva@utoronto.ca]

Giacomina Rossi, Fondazione Istituto di Ricovero e Cura a Carattere Scientifico, Istituto Neurologico Carlo Besta, Milan, Italy [giacomina.rossi@istituto-besta.it]

Martin N. Rossor, Dementia Research Centre, UCL Institute of Neurology, London, UK [m.rossor@ucl.ac.uk]

David Tang-Wai, University Health Network Memory Clinic, Toronto Western Hospital, Toronto, ON, Canada [david.tang-wai@uhn.ca]

David L. Thomas, Neuroradiological Academic Unit, UCL Institute of Neurology, London, UK [d.thomas@ucl.ac.uk]

Hakan Thonberg, Center for Alzheimer Research, Division of Neurogeriatrics, Karolinska Institutet, Stockholm, Sweden [hakan.thonberg@karolinska.se]

Pietro Tiraboschi, Fondazione Istituto di Ricovero e Cura a Carattere Scientifico, Istituto Neurologico Carlo Besta, Milan, Italy [pietro.tiraboschi@istituto-besta.it]

Ana Verdelho, Department of Neurosciences, Santa Maria Hospital, University of Lisbon, Lisbon, Portugal [averdelho@medicina.ulisboa.pt]

Jason D. Warren, Dementia Research Centre, UCL Institute of Neurology, London, UK [jason.warren@ucl.ac.uk]

## Abbreviations

AC: Anterior cingulate rating scale
AT: Anterior temporal rating scale
*C9ORF72*: Chromosome 9 open reading frame 72
CSF: Cerebrospinal fluid
FI: Frontoinsula rating scale
FTD: Frontotemporal dementia
*GRN*: Progranulin
ICC: Intraclass correlation coefficient
*MAPT*: Microtubule-associated protein tau
MTA: Medial temporal atrophy rating scale
OF: Orbitofrontal rating scale
PA: Posterior atrophy rating scale
